# Attachment, Physical Leisure, and Suicidal Ideation in Emerging Adulthood: Evidence from University Students

**DOI:** 10.3390/bs16091674

**Published:** 2026-09-17

**Authors:** Jessica Morales-Sanhueza, Guadalupe Martín-Mora-Parra, Ismael Puig-Amores

**Affiliations:** 1 Facultad de Ingeniería, Universidad Católica de Temuco, Temuco 478000, Chile; jmorales@uct.cl; 2 Carrera de Psicopedagogía, Facultad de Educación, Universidad Autónoma de Chile, Temuco 478000, Chile; 3 Department of Psychology and Anthropology, University Institute for Educational Research and Prospection (INPEX), University of Extremadura, 06071 Badajoz, Spain; ipuigamores@unex.es

**Keywords:** suicidal ideation, attachment styles, physical leisure, emerging adulthood, university students, mental health, sex differences, Chile

## Abstract

University students are particularly vulnerable to mental health problems during emerging adulthood, and suicidal ideation represents a major public health concern. This study examined the relationships among attachment styles, leisure activities, social network use, and suicidal ideation in Chilean university students, considering the role of biological sex. A cross-sectional, descriptive–correlational design was employed with a sample of 1096 students aged 18–29 years. Participants completed measures of suicidal ideation, adult attachment, and leisure activities. Descriptive statistics, chi-square analyses, ANOVA, and binary logistic regression models were conducted. Results revealed a high prevalence of medium-to-high suicidal ideation (55.9%) and insecure attachment styles (63.5%). Women reported higher levels of insecure attachment, a greater risk of suicidal ideation, and lower participation in physical leisure activities than men. Insecure attachment was significantly associated with a higher likelihood of suicidal ideation, whereas participation in physical leisure activities was associated with a lower likelihood of suicidal ideation. Logistic regression analyses confirmed that both attachment style and physical leisure remained significant predictors of suicidal ideation after controlling for sex. No significant associations were found between suicidal ideation and daily internet use or most social network variables. These findings highlight the relevance of relational and behavioral factors in understanding suicidal ideation and underscore the potential value of promoting physical leisure activities and identifying insecure attachment early as areas for further research and intervention in university populations.

## 1. Introduction

### 1.1. Mental Health and Suicidal Ideation in Emerging Adulthood

College students are particularly vulnerable to developing mental health problems during the transition to adulthood (Auerbach et al., 2018; Barrera-Herrera & San Martín, 2021; Tanner & Arnett, 2016). This vulnerability is reflected in the high prevalence of symptoms of anxiety, depression, perceived loneliness, and hopelessness observed during this stage of life (Campbell et al., 2022; Mortier et al., 2018; Rotenstein et al., 2016; Solmi et al., 2022), as well as in the increase in suicidal behaviors and ideation (Granieri et al., 2022; World Health Organization, 2025). The importance of this phenomenon is particularly concerning given that suicide remains one of the leading causes of death among people aged 15 to 29 years worldwide and in Chile (Araneda et al., 2021; World Health Organization, 2025). Suicidal ideation, defined as the desire to die associated with hopelessness and psychological pain, is one of the most important predictors of future suicide attempts and more serious suicidal behaviors among college students (Klonsky et al., 2016, 2018). Therefore, its early identification is a priority for prevention among university students (Franklin et al., 2017).

The high incidence of psychological difficulties observed in this group has been associated with the developmental changes and psychosocial demands of emerging adulthood (Arnett et al., 2014). Arnett (2000) defined this period, which extends from approximately 18 to 29 years of age, as a transitional stage characterized by identity exploration, instability, and a progressive search for autonomy. During this stage, young adults must simultaneously face increasingly complex academic, occupational, economic, social, and emotional demands. Consequently, these circumstances may increase levels of psychological stress, emotional distress, and suicidal ideation in this population (Arnett et al., 2014; Campbell et al., 2022), particularly among young women (Auerbach et al., 2018; Matud et al., 2015).

### 1.2. Attachment Styles and Psychological Vulnerability

Attachment Theory (Bowlby, 1969, 1982) provides a framework for understanding individual differences in emotional regulation, psychological adaptation, and interpersonal relationships, with early caregiving experiences contributing to relatively stable attachment patterns (Bowlby, 1969, 1982; Ainsworth et al., 1978).

Available evidence indicates that secure attachment, characterized by low levels of attachment anxiety and avoidance, is associated with a greater ability to cope with stressful situations, regulate emotions, and maintain satisfactory interpersonal relationships (Guzmán-González et al., 2016; Mikulincer & Shaver, 2018; Salavou et al., 2026). Secure attachment may also act as a protective factor against anxiety, depression, and suicidal ideation (Dagan et al., 2018; X. Wang et al., 2026; Yang et al., 2023; Zortea et al., 2021). In contrast, insecure attachment styles (anxious, avoidant, and disorganized), characterized by high levels of anxiety and/or avoidance, have been consistently associated with greater psychological vulnerability and suicide risk (Maciel et al., 2026; MacNeil et al., 2023; Mikulincer & Shaver, 2019; Soto-Sanz et al., 2025; X. Wang et al., 2026). Likewise, several studies have identified higher levels of attachment anxiety in women and higher levels of attachment avoidance in men (Del Giudice, 2019; Li et al., 2019; Vaillancourt-Morel et al., 2022).

### 1.3. Attachment and Leisure During Emerging Adulthood

Beyond their influence on emotional regulation and mental health, attachment styles may also shape how individuals relate to their social environment and engage in experiences of personal exploration and development. Since Bowlby’s (1988) original formulation, secure attachment has been associated with a greater willingness to explore the environment and establish satisfying interpersonal relationships, whereas insecure styles tend to be characterized by patterns of avoidance, dependence, or difficulties in seeking social support. During emerging adulthood, many of these experiences of exploration, social interaction, and identity construction take place in leisure contexts (Arnett, 2000; Arnett et al., 2014; Caldwell & Witt, 2011). Thus, attachment styles may influence how young people use their free time, participate in recreational activities, and develop meaningful social bonds.

### 1.4. Leisure Activities, Basic Psychological Needs, and Suicidal Ideation

Self-determination theory (Deci & Ryan, 2000; Ryan & Deci, 2017) proposes that the satisfaction of basic psychological needs is associated with well-being and resilience, whereas their frustration is related to psychological vulnerability (Deci & Ryan, 2000; Ryan & Deci, 2017; Ng et al., 2012; Vansteenkiste et al., 2020). From this perspective, leisure activities, understood as the set of voluntary activities carried out during free time (Hills & Argyle, 1998), constitute particularly relevant contexts for satisfying these needs during emerging adulthood (Ou et al., 2025). Various reviews have shown that participation in active and socially inclusive recreational activities (physical leisure) is associated with higher levels of psychological well-being and perceived social support, as well as lower rates of suicidal ideation (Zhou & Sun, 2025). In contrast, leisure patterns characterized by social isolation or a high dependence on digital activities are associated with greater psychological distress and loneliness (Cai et al., 2023; Fancourt et al., 2021; Guo et al., 2024; Sedgwick et al., 2019). Consequently, leisure can be conceptualized as a potential risk or protective factor in relation to suicidal ideation.

Although attachment theory and self-determination theory arise from different theoretical traditions, they both emphasize the importance of interpersonal relationships for psychological development and well-being. From this perspective, participation in leisure activities may constitute a context in which attachment styles facilitate or hinder the satisfaction of the basic psychological needs proposed by self-determination theory, with possible implications for psychological well-being and suicidal ideation.

### 1.5. Social Media Use as a Contemporary Relational Context

Currently, a significant proportion of young people’s interpersonal relationships and leisure activities take place in digital environments. Consequently, social networks have been considered a relevant context for understanding psychological well-being and the risk of suicidal ideation during emerging adulthood (Cai et al., 2023; He et al., 2024). Although these platforms can facilitate social connection and access to interpersonal support, several studies have indicated that certain patterns of problematic use are associated with higher levels of depression, anxiety, negative social comparison, and suicidal ideation (Marchant et al., 2021). Likewise, the literature suggests that attachment styles can influence how young people use these digital environments (Kelly et al., 2019 Vaillancourt-Morel et al., 2022).

### 1.6. Institutional Context

The study was conducted with a sample of students from Universidad Católica de Temuco, a higher education institution located in the Araucanía Region, Chile. The university operates under a competency-based educational model and offers more than 40 professional degree programs, with a total undergraduate enrollment of approximately 13,000 students (57% female and 43% male), predominantly within the emerging adulthood age group.

### 1.7. Objectives and Hypotheses

Despite the existing evidence on the associations among attachment, leisure, social networks, and suicidal ideation, these factors are usually analyzed independently. Few studies have examined these variables jointly during emerging adulthood while also considering the possible role of sex in these relationships.

The present study aimed to analyze the relationship among attachment security/insecurity, leisure activities, and suicidal ideation in young university students, considering the role of sex and exploring the possible influence of social network use.

The following hypotheses were proposed: (i) significant differences were expected to exist according to biological sex, such that women would have a higher prevalence of insecure attachment styles, a higher probability of suicidal ideation and a lower participation in physical leisure activities than men; (ii) insecure attachment styles and patterns of internet use (hours of use and social networks) were expected to be associated with a higher probability of presenting suicidal ideation in young university students; (iii) greater participation in physical leisure activities was expected to be associated with a lower probability of presenting suicidal ideation; and (iv) attachment style and participation in physical leisure activities were expected to contribute significantly to the explanation of suicidal ideation, even after controlling for the effect of sex.

## 2. Materials and Methods

### 2.1. Participants

Non-probabilistic convenience sampling was used, with participants selected based on their accessibility and willingness to participate in the study.

The final sample comprised 1096 young people (N = 1096), of whom 713 were women (64.9%) and 383 were men (35.1%), aged between 18 and 29 years (M = 20.75, SD = 2.21). All participants were students at Catholic University of Temuco (Chile). The sociodemographic characteristics of the participants are presented in Table 1.

### 2.2. Procedure

Prior to data collection, the project was evaluated and approved by the Bioethics and Biosafety Committee of the University of Extremadura (Spain) (Ref. 95/2023). Subsequently, the Catholic University of Temuco was contacted to request authorization and access to the students.

Participants were informed about the objectives of the research, the voluntary nature of participation, and the confidentiality of the data, and informed consent was obtained from all participants.

Data collection was conducted in the classroom context in a single session. After coordinating the date and time of administration with each participating group, the researchers visited the classroom to administer the questionnaires. Students accessed the questionnaires through a QR code that linked to a form on Google Forms and completed the instruments individually using their mobile devices or computers.

During administration, the researchers remained present to clarify any questions without influencing participants’ responses. The data was collected and organized automatically, allowing subsequent export for statistical analysis.

### 2.3. Instruments

A brief sociodemographic questionnaire designed ad hoc was administered to collect information on their age, biological sex, preferred social networks, time spent using the internet, and leisure activities. To assess leisure activities, participants completed an open-ended item regarding the activities they usually participated in during their free time (“What do you usually do in your free time?”). Respondents were allowed to report an unlimited number of activities; however, no data was collected regarding the frequency or duration of these activities.

Based on participants’ open-ended responses, leisure activities were classified according to the multidimensional framework established by H.-X. Wang et al. (2002) and Karp et al. (2006) and further conceptualized by Bielak (2010). This framework organizes leisure activities into three interrelated domains: (a) physical leisure (activities involving bodily movement and energy expenditure, such as running or fishing), (b) cognitive leisure (activities oriented toward information processing and mental stimulation), and (c) social leisure (activities based on interpersonal interaction and community integration).

The following instruments were also used:

Suicidal Ideation Frequency Inventory (FSII), adapted into Spanish by Sánchez-Álvarez et al. (2020).

This instrument assesses the frequency of suicidal thoughts during the previous 12 months using five items with a 5-point Likert-type response format (1 = never, 5 = almost every day). The total possible score ranges from 5 to 25, with higher scores indicating a higher frequency of suicidal ideation. The Spanish version has shown adequate psychometric properties, with a Cronbach’s alpha coefficient of 0.89 and an internal consistency coefficient of 0.94 (Sánchez-Álvarez et al., 2020).

Experience in Close Relationships Scale—Revised (ECR-R) (Alonso-Arbiol et al., 2007).

This self-report instrument assesses adult attachment and is based on two dimensions: anxiety and avoidance in affective relationships. The Spanish version of the questionnaire was used in this study. The questionnaire consists of 36 items distributed across the two dimensions using a 7-point Likert-type response format (1 = strongly disagree, 7 = strongly agree). Based on these dimensions, participants can be classified into four adult attachment styles: secure, avoidant, anxious, and disorganized. The Spanish version has demonstrated adequate reliability indices, with a Cronbach’s alpha coefficient of 0.87 for the avoidance subscale and Cronbach’s alpha coefficients of 0.85 for the anxiety subscale (Alonso-Arbiol et al., 2007).

### 2.4. Study Design

The study employed a cross-sectional, descriptive–correlational design.

A sensitivity analysis was performed using G*Power 3.1. The analysis was based on the final sample size (N = 970), a significance level of α = 0.05, a two-tailed test, and a target statistical power of 0.80. Under these parameters, the minimum detectable odds ratio was 0.798 (equivalent to an odds ratio of approximately 1.25 in the opposite direction). The dependent variable was suicidal ideation, operationalized at two levels (low suicidal ideation and medium/high suicidal ideation). For the construction of the initial categories, extreme-core criteria were used, establishing cut-off points at ±1 standard deviation from the mean (Aiken & West, 1991). Subsequently, the variable was dichotomized into two groups (low suicidal ideation vs. medium/high suicidal ideation) to facilitate the estimation of binary logistic regression models. Independent variables included age, biological sex, attachment style, and participation in leisure activities.

### 2.5. Data Analysis

Statistical analyses were performed using SPSS (Statistical Package for the Social Sciences) version 26 (IBM Corp, 2019).

The effective sample size for the inferential analyses was reduced from 1096 to 970 participants because of missing values in some of the variables included in the statistical models (suicidal ideation, attachment style or physical leisure). A listwise deletion procedure was applied to handle this missing data (Allison, 2001; Enders, 2010), so that only cases with complete information on all the variables included in each model were analyzed. Although this procedure reduced the effective sample size, it maintained the internal coherence of the analyses and minimized potential biases associated with imputing categorical or highly asymmetric variables.

First, descriptive analyses were performed to characterize the study variables (biological sex, suicidal ideation, attachment style, and leisure activities), using frequencies, percentages, means, and standard deviations.

The total score on the Suicidal Ideation Frequency Inventory (FSII) was classified into three ordinal categories of suicidal ideation: low (0), medium (1), and high (2). Subsequently, this variable was dichotomized into two groups (low suicidal ideation = 0; medium/high suicidal ideation = 1) for the binary logistic regression models.

For the multivariate logistic regression analyses, attachment style was also recoded into a dichotomous variable distinguishing secure and insecure attachment, coded as 0 and 1, respectively. The four attachment profiles (secure, anxious, avoidant, and disorganized) were retained for the bivariate analyses, whereas the dichotomous classification was used in the multivariate models to examine the overall association between attachment security/insecurity and suicidal ideation.

Bivariate analyses were then performed to examine associations among the study variables. For categorical variables, Pearson’s chi-square test was used, with effect size estimated using Cramer’s V statistic. For quantitative variables, a one-way analysis of variance (ANOVA) was applied, after checking the homogeneity of variances using Levene’s test.

Finally, binary logistic regression models were performed to analyze the joint effects of the independent variables on the probability of presenting medium or high levels of suicidal ideation. The models estimated B coefficients, standard errors, Wald statistics, odds ratios (ORs), and 95% confidence intervals.

Furthermore, to assess the robustness of the findings derived from the dichotomization of attachment into secure and insecure categories, a sensitivity analysis was performed using a multiple linear regression model. This analysis was conducted with continuous scores for suicidal ideation, attachment anxiety, and attachment avoidance, allowing for the examination of associations between the original continuous variables and minimizing the potential loss of information associated with categorical classification.

## 3. Results

### 3.1. Descriptive Statistics

A descriptive analysis of the sample and the main study variables was first conducted. The sample was predominantly composed of women (65.1%), while men represented 34.9%.

In terms of suicidal ideation, 44.1% of the participants were in the low-risk group, whereas 55.9% were in the medium- or high-risk groups.

Regarding attachment style, approximately one-third of the students showed secure attachment (36.5%), whereas 63.5% presented insecure attachment.

Regarding leisure activities, physical leisure (running, walking, going to the gym, dancing, and fishing) was reported by 31.8% of the sample. Cognitive leisure (reading, watching series or films, and playing video games) was reported by 32.9%, whereas social leisure (interacting with friends, family, or a partner) was reported by 5%. Finally, rest-related leisure (sleeping, listening to music, and napping) was reported by 3.4%. The percentages do not sum to 100% because participants could report more than one leisure activity.

### 3.2. Association Between Sex and Attachment Style

A differential distribution of attachment styles was observed by biological sex. Specifically, men showed a higher proportion of secure attachment (39.7%) than women (34.8%), as well as a lower relative proportion of insecure styles. In contrast, women showed a higher proportion of anxious (16.8% vs. 11% in men) and disorganized (17.5% vs. 9.9% in men) attachment, whereas the difference in avoidant attachment was more moderate (30.9% in women vs. 39.2% in men) (Table 2).

These patterns suggest a differential profile by sex, characterized by greater attachment security among men and a greater representation of insecure styles among women, particularly those related to anxiety and disorganization.

Pearson’s chi-square test confirmed a significant association between the two variables (χ^2^ = 24.098, df = 3, *p* < 0.001), with a small-to-moderate effect size (Cramer’s V = 0.148), indicating that, although the association was statistically significant, its magnitude was limited.

### 3.3. Association Between Sex and Leisure Activities

Differences in participation in leisure activities by biological sex were analyzed. No significant associations were observed for cognitive, social, or rest-related leisure (*p* > 0.05 in all cases), indicating a similar distribution between men and women for these types of activities.

In contrast, physical leisure showed a significant association with sex (χ^2^ = 83.115, df = 1, *p* < 0.001), with a moderate effect size (Cramer’s V = 0.275). As shown in Table 3, men had higher participation in physical leisure activities (49.3%) than women (22.4%), whereas women had a higher proportion in the non-participation group (77.6% compared with 50.7% among men).

These results indicate a clear sex difference in participation in physical leisure activities, with greater involvement among men.

### 3.4. Association Between Sex, Attachment, and Suicidal Ideation

To identify the factors associated with suicidal ideation in the students, differences in suicidal ideation according to sex and attachment style were first examined.

Sex was significantly associated with suicidal ideation. Specifically, men were more represented in the low-risk group and less represented in the high-risk group, whereas women showed the opposite pattern (χ^2^ = 35.21, *p* < 0.001; Cramer’s V = 0.191, *p* < 0.001). Thus, in this sample, suicidal ideation differed significantly by sex (Table 4).

Similarly, attachment style showed a significant association with suicidal ideation (χ^2^ = 50.70, df = 6, *p* < 0.001; Cramer’s V = 0.229, *p* < 0.001). Anxious and disorganized styles were more frequent in the medium- and high-risk groups, whereas secure attachment predominated in the low-risk group, suggesting that insecure attachment patterns may be associated with greater vulnerability to suicidal ideation (Table 5).

### 3.5. Association Between Leisure Activities and Suicidal Ideation

To examine factors associated with suicidal ideation in university students and their possible inverse relationships, differences in suicidal ideation according to sex, attachment, and participation in different leisure activities were evaluated. No significant associations were observed for cognitive leisure (χ^2^ = 1.50, *p* = 0.473), social leisure (χ^2^ = 3.40, *p* = 0.183), or rest-related leisure (χ^2^ = 0.50, *p* = 0.781).

In contrast to the above, physical leisure (running, walking, going to the gym, dancing, and fishing) showed a significant relationship with suicidal ideation (χ^2^ = 25.85, df = 2, *p* < 0.001) (Table 6).

These results suggest that participants who engaged in physical leisure were more represented in the low-risk group and less represented in the high-risk group, indicating a possible association with a lower likelihood of suicidal ideation.

### 3.6. Association Between Social Networks and Suicidal Ideation

The association between social network use and suicidal ideation was examined by differentiating among visual, textual, and community-based networks. Visual (χ^2^ = 0.46, *p* = 0.795) and community-based (χ^2^ = 0.76, *p* = 0.686) networks showed no significant associations. Textual networks showed a significant linear pattern (linear-by-linear association = 7.59, *p* = 0.006), although the overall chi-square test did not reach conventional statistical significance (χ^2^ = 9.01, df = 2, *p* = 0.061) (Table 7).

### 3.7. Hours of Internet and Suicidal Ideation

To explore possible risks associated with digital use, we evaluated whether the number of hours spent on the internet each day was associated with suicidal ideation using ANOVA. Levene’s test indicated homogeneity of variances (statistic = 0.679, df = 2.967, *p* = 0.507). Likewise, the ANOVA showed no significant differences among the risk groups (F = 0.955, df = 2.967, *p* = 0.385), indicating that the number of hours spent on the internet was not significantly associated with suicidal ideation (Table 8).

### 3.8. Binary Logistic Regression Analysis

Finally, to examine whether physical leisure and attachment style contributed to the association between sex and suicidal ideation, binary logistic regression models were performed. The first step assessed whether sex (male = 0; female = 1) predicted participation in physical leisure (no = 0; yes = 1) (Table 9).

The results of the binary logistic regression indicated that sex was a significant predictor of participation (B = −1.214, SE = 0.136, Wald = 79.657, *p* < 0.001). Using men as the reference category, women were significantly less likely to participate in leisure physical activities (OR = 0.297).

Physical leisure was subsequently included in the model together with sex to predict binary suicidal ideation (suicidal ideation/non-suicidal ideation). The results indicated that participation in physical leisure was significantly associated with lower odds of suicidal ideation. In addition, women had 1.88 times higher odds of suicidal ideation than men (OR = 1.883) (Table 10).

Finally, a binary logistic regression model was performed to examine the associations of sex, physical leisure, and attachment with suicidal ideation. This analysis did not include variables related to the preferred social network (textual, visual, community, etc.) or the number of hours of internet use, as these variables did not show significant associations with suicidal ideation in the previous analyses (Table 11).

A valid final regression model was obtained (*p* < 0.001), with a Nagelkerke R^2^ of 0.085, correctly classifying 63.2% of the cases. The Hosmer–Lemeshow goodness-of-fit test yielded a χ^2^ value of 3.900 (*p* = 0.690). These findings suggest that both physical leisure and attachment are relevant variables for understanding suicidal ideation among young university students. Although the final model showed a relatively modest Nagelkerke, the results identified significant associations that may be relevant for understanding suicidal ideation in this population. Specifically, participation in physical activities was associated with a lower likelihood of suicidal ideation, even after controlling for sex, whereas insecure attachment was associated with a higher probability of suicidal ideation, reinforcing the importance of affective relationship patterns as a factor of vulnerability.

### 3.9. Further Analysis

As a sensitivity analysis, a multiple linear regression was performed using the continuous dimensions of attachment anxiety and avoidance from the ECR-R as predictors and the total suicidal ideation score as the outcome variable. The model was statistically significant, F = 44.38, *p* < 0.001, explaining 7.5% of the variance in suicidal ideation (R^2^ = 0.075). Both attachment anxiety (β = 0.213, *p* < 0.001) and attachment avoidance (β = 0.146, *p* < 0.001) were positively associated with suicidal ideation, with attachment anxiety showing a stronger association (Table 12).

## 4. Discussion

First, the results showed a high prevalence of suicidal ideation among the young people in the sample. These findings are consistent with previous research identifying university students as a group that is particularly vulnerable to mental health problems compared with the general population and non-university youth (Barrera-Herrera & San Martín, 2021; Franzoi et al., 2021; Granieri et al., 2021, 2022). Likewise, a high prevalence of insecure attachment styles was observed, consistent with previous studies conducted in university populations (Martín-Mora-Parra et al., 2026; Masapanta Solís & Nuñez Núñez, 2023; Morales-Sanhueza & Martín-Mora-Parra, 2024; Mayorga-Parra & Vega Falcón, 2021). Additionally, the results showed that, among the different leisure activities evaluated, physical leisure and cognitive leisure were the most frequent modalities among the participants.

The findings also supported the first research hypothesis, showing significant differences according to biological sex. Compared with men, women had a higher prevalence of insecure attachment styles, a higher probability of belonging to the medium- and high-risk groups for suicidal ideation, and lower participation in physical leisure activities. In particular, women more frequently exhibited insecure attachment patterns, especially those characterized by high levels of anxiety. These results are consistent with previous research identifying higher frequencies of anxious and disorganized attachment among young women (Del Giudice, 2019; Morales-Sanhueza & Martín-Mora-Parra, 2024; Morales-Sanhueza et al., 2024; Weber et al., 2022). The literature indicates that these forms of attachment insecurity are generally associated with greater difficulties in regulating negative emotions and with a tendency toward rumination, thereby increasing psychological vulnerability to stressful situations (Ando et al., 2020; Sánchez-Cabada et al., 2022; Weber et al., 2022). This may help explain the higher proportion of women in the medium- and high-risk groups for suicidal ideation, whereas an inverse distribution was observed among men.

The analyses also indicated that sex was associated with participation in physical leisure activities. Men showed a higher probability of participating in this type of activity than women. These results are consistent with the previous literature documenting sex differences in physical activity levels, particularly among university students, where women tend to report lower participation in physical activities (Espada et al., 2023). One possible explanation is that women may show lower levels of intrinsic motivation toward physical activity than men and may therefore be less likely to engage in this type of activity during their leisure time (Asfaw et al., 2020; Lázaro-Pérez et al., 2023; Miranda-Mendizabal et al., 2019; Rodríguez-Romo et al., 2022).

Regarding the second hypothesis, the findings provided partial support. First, the results of the study show that insecure attachment styles were significantly associated with a higher likelihood of presenting suicidal ideation. Accumulating evidence suggests that attachment insecurity is an important vulnerability factor for the appearance of suicidal thoughts and other indicators of deteriorating mental health (Dienst et al., 2023; Granieri et al., 2022; Green et al., 2021; Potard et al., 2020). In addition, the results showed that attachment styles characterized by high levels of anxiety were more frequent in the medium- and high-risk groups for suicidal ideation, whereas secure attachment predominated in the low-risk group. Importantly, an additional sensitivity analysis conducted using the original continuous dimensions of attachment anxiety and attachment avoidance, together with continuous suicidal ideation scores, yielded a consistent pattern of results. Both attachment dimensions were positively associated with suicidal ideation, suggesting that the observed association between attachment insecurity and suicidal ideation was not solely attributable to the dichotomization of attachment and supporting the robustness of the primary findings. One possible explanation for this association is that attachment anxiety may reduce the use of adaptive coping strategies when individuals face complex or stressful situations. This may increase feelings of hopelessness and meaninglessness, with suicidal ideation potentially emerging as an escape from psychological distress (Holdaway et al., 2018; Law & Tucker, 2018; Yu & Zhao, 2023).

Unexpectedly, no significant associations were observed between social network use, the hours of internet use, and suicidal ideation. This finding contrasts with previous evidence linking problematic use of the internet and social network use to a higher likelihood of mental health problems and suicidal ideation (Bersani et al., 2022; Kennard et al., 2025; Ma et al., 2025; Marano et al., 2025; Nesi et al., 2022; Xiao et al., 2025).

The results also showed a significant association between physical leisure and suicidal ideation. Specifically, participation in physical leisure activities was associated with lower odds of suicidal ideation, supporting the third hypothesis. Consistent with this finding, previous research has documented associations between physical activity and higher levels of meaning in life, self-efficacy, and life satisfaction, all of which are relevant to the understanding of suicidal ideation (Guo et al., 2024; Ou et al., 2025; Zhou & Sun, 2025). These findings highlight the importance of the context in which physical activity takes place. In particular, various studies have indicated that physical activity performed during leisure time may have a stronger effect on mental health risk factors and suicidal ideation (Elsden et al., 2022; Fancourt et al., 2021, 2023; Rodríguez-Romo et al., 2022; Teychenne et al., 2026).

Taken together, these findings support considering attachment style and leisure activities jointly in the study of suicidal ideation. Although they represent different aspects of psychological functioning, both are related to emotional regulation, interpersonal engagement, and psychological well-being during emerging adulthood. Attachment may influence how young people engage with social environments, while leisure contexts may provide opportunities to satisfy basic psychological needs such as autonomy, competence, and relatedness. Thus, attachment and leisure can be understood as complementary relational and behavioral factors that may contribute to differential vulnerability to suicidal ideation.

Another supported fourth research hypothesis was that both attachment style and participation in physical leisure activities contributed significantly to the explanation of suicidal ideation, even after accounting for biological sex. The inclusion of both variables reduced the magnitude of the association between sex and suicidal ideation, although sex remained statistically significant. Thus, the results suggest that the observed differences between men and women may be partially understood in relation to relational and behavioral factors.

In particular, the results indicate that insecure attachment styles were associated with a higher likelihood of suicidal ideation, consistent with evidence linking attachment insecurity to greater difficulties in emotional regulation, interpersonal disconnection, and suicide risk among young university students (Dienst et al., 2023; Granieri et al., 2022; Green et al., 2021; Martín-Mora-Parra et al., 2026; Morales-Sanhueza & Martín-Mora-Parra, 2024). These results reinforce the relevance of affective relationship patterns as an important vulnerability factor for suicidal ideation (Martín-Mora-Parra et al., 2026).

Similarly, participation in physical leisure activities was associated with a lower likelihood of suicidal ideation, even after controlling for the effect of sex, supporting evidence highlighting the potential buffering role of physical activity (Firth et al., 2020; Guo et al., 2024; Miranda-Mendizabal et al., 2019; Ou et al., 2025; Vidal-Arenas et al., 2022). Additionally, the coexistence of insecure attachment styles and lower participation in physical leisure activities may partially explain the higher risk of suicidal ideation observed among women in the sample. The previous literature has indicated that various emotional vulnerability factors associated with insecure attachment may constitute risk factors for suicidal ideation and, in turn, may be related to a lower probability of participation in physical leisure activities (Dienst et al., 2023; Espada et al., 2023; Green et al., 2021; Pulido et al., 2021; Rodríguez-Romo et al., 2022; Romero-Parra et al., 2023; Sevil-Serrano et al., 2017). However, given the cross-sectional nature of the study, these findings should be interpreted cautiously because causal relationships cannot be established.

Finally, this finding highlights the importance of simultaneously considering relational and behavioral factors when examining suicidal ideation among young university students. The promotion of physical leisure activities and the early identification of insecure attachment styles may represent relevant areas for further research and intervention, particularly among students experiencing greater emotional difficulties. Future research could further examine the association between physical leisure and suicidal ideation, particularly among women, given previous evidence of relevant associations between participation in physical leisure activities and mental health outcomes in this group (Kim et al., 2019; Guo et al., 2024).

## 5. Limitations

One limitation of this research is its cross-sectional design, which prevents causal inferences. Longitudinal research could provide a complementary perspective and help clarify the temporal relationships among the variables.

Additionally, the sample included only university students; therefore, the findings cannot be assumed to be representative of all young people. Future research should broaden the scope of the study by including samples composed of children, adolescents, and adults from the general population.

Furthermore, physical leisure was evaluated globally, without distinguishing between specific modalities. Consequently, future research should examine whether distinct types of activity trigger different neurobiological or psychosocial mechanisms in mitigating suicidal ideation.

Another limitation concerns the dichotomization of attachment into secure and insecure categories, which may have reduced the variability captured by the original attachment dimensions and obscured differences between specific insecure attachment profiles. However, sensitivity analyses based on the continuous dimensions of attachment anxiety and avoidance yielded convergent results, further supporting the strength of the observed associations.

Finally, all variables evaluated were measured using self-report instruments, which may introduce biases related to social desirability, recall errors, or underreporting of certain experiences and behaviors. Similarly, the use of convenience sampling limits the generalizability of the findings to other university populations and sociocultural contexts. Finally, other potentially relevant variables associated with suicidal ideation, such as mental health history, social support, stressful life experiences, or substance use, were not included and may influence the observed associations.

## 6. Conclusions

The results of this research provide relevant evidence regarding the role of attachment, physical leisure, and sex in the understanding of suicidal ideation in the Chilean university population. Specifically, the findings show that participation in physical leisure activities was associated with a lower probability of suicidal ideation and was also associated with the sex of the participants, contributing to a partial understanding of the differences observed between men and women in this phenomenon. Thus, lower participation in physical leisure activities may represent one relevant factor in explaining sex differences in suicidal ideation.

Likewise, insecure attachment styles, particularly those characterized by high levels of anxiety, were associated with a higher probability of suicidal ideation, reinforcing the relevance of attachment patterns as a vulnerability factor.

These findings contribute to a broader understanding of the psychosocial factors associated with suicidal ideation in university populations by integrating behavioral and relational variables within the same explanatory model.

Based on these findings, the importance of designing preventive strategies in university contexts is highlighted. Participation in physical leisure activities may represent a relevant area for further research and intervention, particularly among women, who showed lower levels of participation in this type of activity and a higher prevalence of suicidal ideation in association with attachment and emotional factors.

Finally, future research should further examine the interaction among these factors, together with other emotional and contextual variables, to better understand more precisely the relationships underlying the differences observed by sex.

## Figures and Tables

**Table 1:** Sociodemographic characteristics.

Variable	Category/Statistic	n	%
Sex	Men	383	35.1
	Women	713	64.9
Age	M (SD)	20.93 (2.84)	—
	Range	18–29	—

**Table 2:** Distribution of attachment style according to sex.

Attachment Style	Men	Women
Anxious	42 (11%)	120 (16.8%)
Disorganized	38 (9.9%)	125 (17.5%)
Avoidant	150 (39.2%)	220 (30.9%)
Secure	152 (39.7%)	248 (34.8%)
Total	383 (100%)	713 (100%)

**Table 3:** Distribution of participation in physical leisure by sex.

Physical Leisure	Men	Women	Total
Does not do the activity	194 (50.7%)	553 (77.6%)	747
Yes, does the activity	189 (49.3%)	160 (22.4%)	349
Total	383 (100%)	713 (100%)	1096

**Table 4:** Suicidal ideation by sex.

Suicidal Ideation	Men	Women	Total
Low	200	228	428
Medium	100	202	302
High	59	181	240
Total	359	611	970

**Table 5:** Suicidal ideation according to attachment style.

Suicidal Ideation	Anxious	Disorganized	Avoidant	Secure	Total
Low	47	30	152	199	428
Medium	49	39	111	103	302
High	43	53	77	67	240
Total	139	122	340	369	970

**Table 6:** Suicidal ideation according to participation in physical leisure.

Suicidal Ideation	Not Active	Yes, Active	Total
Low	251	177	428
Medium	212	90	302
High	185	55	240
Total	648	322	970

**Table 7:** Suicidal ideation according to use of textual social networks.

Suicidal Ideation	I Do Not Use	Application
Low	337	90
Medium	244	58
High	210	30
Total	791	178

**Table 8:** Mean and standard deviation of hours spent on the internet by level of suicidal ideation.

Suicidal Ideation	N	Medium	Standard Deviation	Typical Error
Low	428	4.25	2.43	0.12
Medium	302	4.50	2.53	0.15
High	240	4.39	2.22	0.14
Total	970	4.36	2.41	0.08

**Table 9:** Binary logistic regression of sex on physical leisure.

Variable	B	SE	Wald	*p*-Value	OR	95% CI	
Biological sex	−1.214	0.136	79.657	<0.001 *	0.297	0.227	0.388
Constant	−0.026	0.102	0.065	0.798	0.974	—	—

Note: Sex was coded as 0 = male and 1 = female. OR = odds ratio; CI = confidence interval; (*) = significance.

**Table 10:** Binary logistic regression of sex and physical leisure on suicidal ideation.

Variable	B	SE	Wald	*p*-Value	OR	95% CI	
Biological sex	0.633	0.141	20.267	<0.001 *	1.883	1.429	2.479
Physical leisure	−0.490	0.144	11.566	0.001 *	0.613	0.462	0.813
Constant	0.007	0.128	0.003	0.957	1.007	—	—

Note: Biological sex was coded as 0 = male and 1 = female. Physical leisure was coded as 0 = no engagement in physical leisure activities and 1 = engagement in physical leisure activities. OR = odds ratio; CI = confidence interval; (*) = significance.

**Table 11:** Binary logistic regression with sex, physical leisure, and attachment as predictors of suicidal ideation.

Variable	B	SE	Wald	*p*-Value	OR	95% CI	
Biological sex	0.629	0.142	19.631	<0.001 *	1.877	1.421	2.479
Physical leisure	−0.463	0.146	10.118	0.001 *	0.629	0.473	0.837
Attachment	0.613	0.137	20.042	<0.001 *	1.846	1.412	2.415
Constant	−0.376	0.156	5.837	0.016 *	0.687	—	—

Note: Sex was coded as 0 = male and 1 = female. Physical leisure was coded as 0 = no and 1 = yes. Attachment was coded as 0 = secure and 1 = insecure. Suicidal ideation was coded as 0 = non-suicidal ideation and 1 = suicidal ideation. OR = odds ratio; CI = confidence interval; (*) = significance.

**Table 12:** Sensitivity analysis: Multiple linear regression of suicidal ideation, attachment anxiety, and attachment avoidance (ECR-R).

Variable	B	SE	β	t	*p*-Value	95%CI
Attachment avoidance	0.504	0.102	0.146	4.959	0.000 *	0.305–0.704
Attachment anxiety	0.651	0.090	0.213	7.207	0.000 *	0.473–0.828
Constant	5.645	0.421		13.397	0.000 *	4.818–6.472

Note: CI = confidence interval; (*) = significance.

## Data Availability

The data presented in this study are available upon request from the corresponding author due to ethical approval requirements.
